# Once‐Weekly Insulin Efsitora Alfa Versus Once Daily Insulin in Patients With Type 2 Diabetes: A Systematic Review and Meta‐Analysis

**DOI:** 10.1002/edm2.70126

**Published:** 2025-10-28

**Authors:** Harris Mehmood, Moaz Alowami, Thel Su Hlaing, Khant Nyi Zaya, Yousrah Uraiby, Ahmed Muaaz Alam, Abubakr Adala, Osama Ikram, Syed Faisal Ali, Muzammil Farhan, Eeshal Zulfiqar, Mushood Ahmed, Raheel Ahmed, Ammara Naeem

**Affiliations:** ^1^ Department of General Medicine Alexandra Hospital Worcestershire UK; ^2^ Faculty of Medicine Libyan International Medical University Benghazi Libya; ^3^ Department of Medicine Nevill Hall Hospital Abergavenny UK; ^4^ Department of Medicine Velindre Cancer Centre Hospital Cardiff UK; ^5^ Department of Medicine University Hospitals Birmingham Birmingham UK; ^6^ Faculty of Medicine Masaryk Brno Czech Republic; ^7^ Cardiology Department Glenfield Hospital Leicester UK; ^8^ Department of Endocrinology and Diabetes South Tees NHS Trust Middlesbrough UK; ^9^ Royal Devon University Healthcare NHS Foundation Trust Exeter UK; ^10^ Imperial College London London UK; ^11^ Department of Medicine Dow University of Health Sciences Karachi Pakistan; ^12^ Department of Medicine Rawalpindi Medical University Rawalpindi Pakistan; ^13^ Newcastle University Newcastle upon Tyne UK; ^14^ Sunderland University Sunderland UK; ^15^ Croydon University Hospital NHS Trust South West London UK

**Keywords:** basal insulin Fc, daily basal insulin, diabetes, efsitora, type‐2 diabetes, weekly insulin

## Abstract

**Background:**

Once‐daily basal insulin is widely used in the management of type 2 diabetes, but poor adherence to daily injections often impairs glycaemic control. Once‐weekly efsitora alfa may overcome these limitations, but pooled data assessing its comparative efficacy and safety remain limited.

**Methods:**

PubMed/MEDLINE, Google Scholar, and the Cochrane Library were searched up to July 2025 for RCTs comparing once‐weekly efsitora with once daily insulin in adults with T2D. Weighted mean differences (MDs), odds ratios (ORs), and risk ratios (RRs) were pooled using a random‐effects model, and results were reported with 95% confidence intervals.

**Results:**

Six RCTs comprising 3967 participants were included. There were no significant differences between once‐weekly efsitora and daily insulin in change in HbA1c (MD –0.04; 95% CI –0.10 to 0.02; *p* = 0.15), change in fasting plasma glucose (MD 1.94 mg/dL; 95% CI –2.98 to 6.86; *p* = 0.44), proportion of patients achieving HbA1c < 7%, change in body weight, or time below range. Efsitora was associated with an increase in time in range (MD 0.80 percentage points; 95% CI 0.09 to 1.52; *p* = 0.03) and a reduction in time above range (MD –1.45 percentage points; 95% CI –2.87 to −0.02; *p* = 0.05). The risk of treatment‐emergent adverse events (TEAEs) was higher with efsitora (RR 1.13; 95% CI 1.05 to 1.20; *p* = 0.0004), whereas serious adverse events, hypersensitivity reactions, injection‐site reactions, and hypoglycaemia events were comparable between the two groups.

**Conclusion:**

Once‐weekly efsitora provides comparable glycaemic control and improved time‐in‐range compared to daily insulin, although with a higher rate of TEAEs.

## Graphical abstract

Central illustration summarising the findings of our meta‐analysis.

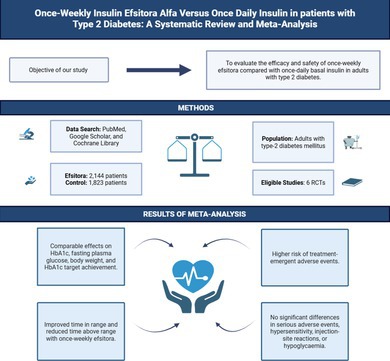

## 1 Introduction

Diabetes remains a major contributor to global mortality and continues to impose a substantial burden on healthcare systems worldwide [1, 2, 3]. According to the Global Burden of Disease (GBD) 2021 report, over 500 million individuals were living with diabetes globally in 2021 [4]. Type 2 diabetes accounts for more than 90% of all cases, and its prevalence continues to rise, primarily driven by increasing rates of obesity and sedentary lifestyles [4, 5, 6, 7].

Long‐acting (basal) insulin remains an essential component of therapeutic strategies in patients with type 2 diabetes who do not achieve adequate glycaemic control with oral or non‐insulin injectable agents. However, practical barriers to its use often undermine the effectiveness of once‐daily insulin. Many patients experience injection phobia or significant discomfort with the daily injections, which negatively impacts adherence and promotes treatment discontinuation [8]. In addition, the requirement for strictly timed daily injections necessitates considerable lifestyle adjustments and imposes a sustained treatment burden [6]. These issues contribute to suboptimal glycemic control, as evidenced by studies showing that fewer than 30% of patients treated with daily insulin achieve target HbA1c levels of < 7% after 1 year of therapy [8, 9, 10, 11].

In this context, the development of once‐weekly insulin analogues represents a major advancement, aiming to address the limitations of daily insulin therapy. Insulin efsitora alfa (basal insulin Fc, BIF or LY3209590) is a novel basal insulin analogue engineered for once‐weekly subcutaneous administration [12, 13]. It functions as an insulin receptor agonist, binding to and activating the insulin receptor to facilitate glucose uptake and suppress hepatic glucose production. To achieve prolonged activity, efsitora incorporates a modified Fc‐fusion structure that extends its half‐life to approximately 15–16 days, supporting a flat and sustained pharmacodynamic profile with minimal fluctuations in circulating insulin levels. These modifications slow both absorption and systemic clearance, thereby enabling stable glycaemic control while substantially reducing injection frequency [12, 13].

Randomised controlled trials have consistently demonstrated that once‐weekly insulin efsitora alfa achieves HbA1c reductions comparable to those of daily insulin (glargine or degludec), while also reducing hypoglycemia risk, limiting dose adjustments, and simplifying titration [14, 15]. Although a previous meta‐analysis reported non‐inferior glycaemic efficacy, it also suggested a higher rate of treatment‐emergent adverse events [16]. Since then, several additional phase III trials have been published, substantially expanding the available evidence base [17, 18]. In this context, the present meta‐analysis offers the most up‐to‐date and comprehensive synthesis of randomised evidence, not only confirming the glycaemic efficacy of once‐weekly efsitora but also providing a detailed assessment of clinically relevant outcomes. By incorporating these newly available data and conducting subgroup analyses to explore sources of heterogeneity, this study refines our understanding of the therapeutic potential of once‐weekly efsitora and its role in optimising long‐term diabetes management.

## 2 Methods

We conducted this systematic review and meta‐analysis using the guidelines established by the Preferred Reporting Items for Systematic Review and Meta‐Analysis (PRISMA) [19]. The study was registered in the PROSPERO database: CRD420251130055. Because only previously published, de‐identified data were used, ethical approval was not required for this study.

### 2.1 Data Sources and Search Strategy

An independent search of PubMed/MEDLINE, Google Scholar, and Cochrane Library was carried out by two independent reviewers (EZ and MA) to include studies from their inception through July 2025. The full search strategies are provided in Table S1. In order to ensure all relevant studies were included, the reference lists of pertinent articles, including previous systematic reviews and meta‐analyses, were also screened manually to identify any additional eligible studies not retrieved through the electronic search.

### 2.2 Study Selection and Eligibility Criteria

Following the literature search, all studies were imported to Rayyan (https://www.rayyan.ai; accessed on 31 July 2025). Duplicates were identified and removed. The remaining articles were reviewed independently by two authors (HM and TSH), based on their titles and abstracts, with further full‐text reviews. Any conflicts were resolved through discussion with a third reviewer (EZ).

The studies were eligible for our systematic review and meta‐analysis if they: (1) were RCTs; (2) included patients with type 2 diabetes mellitus (T2DM); (3) had adult male or female participants who were at least 18 years old; (4) evaluated weekly efsitora alfa; and (5) compared weekly efsitora against daily insulin (degludec or glargine).

We excluded studies if they: (1) involved patients with type 1 diabetes, gestational diabetes, or secondary forms of diabetes; (2) enrolled individuals who had undergone metabolic surgery or had severe comorbid conditions; (3) had non‐randomised designs such as retrospective studies, pooled analyses, case reports, conference abstracts, or letters to the editor; and (4) preclinical studies or trials conducted in animals or healthy human participants.

### 2.3 Data Extraction, Outcomes, and Quality Assessment

Three reviewers (KNZ, SFA, and YU) independently extracted data from each included trial. The following variables were extracted: study name, year of publication, study arms, continuous glucose monitoring (CGM) device used, follow‐up duration, total sample size, mean age and sex of the participants, duration of diabetes, baseline body mass index (BMI in kg/m^2^), body weight (kg), glycated haemoglobin (HbA1c, %), waist circumference (cm), and fasting plasma glucose (FPG in mg/dL).

The primary efficacy outcomes were change in HbA1c and change in fasting plasma glucose from their respective baselines. Secondary efficacy outcomes included the proportion of subjects achieving HbA1C of < 7% and change in body weight, time blood glucose was within, below, and above the target range. The primary safety outcomes were treatment‐emergent adverse events and serious adverse events. Secondary safety outcomes included participants experiencing at least one hypoglycemic alert value, participants experiencing at least one clinically significant hypoglycemic event, participants experiencing at least one severe hypoglycemic event, hypersensitivity reactions, and injection site reactions.

Risk of bias was assessed using Version 2 of the Cochrane Risk of Bias tool for RCTs [20]. The trials were scored as high, with some concerns, or low risk of bias according to the assessment of 5 domains: randomization, deviations from intended variation, missing outcome data, measurement of outcome, and selection of reported results. Traffic‐light and summary plots were created using the Robvis visualisation tool [21].

### 2.4 Statistical Analysis

All statistical analyses were performed using Review Manager (RevMan) version 5.4 (The Nordic Cochrane Centre, Copenhagen, Denmark). For each outcome, pooled mean differences (MD), odds ratios (ORs), or risk ratios (RRs) with corresponding 95% confidence intervals (CIs) were calculated. A random effects model based on the DerSimonian and Laird method was used to account for potential heterogeneity among studies [22]. Forest plots were created to visually present the pooled estimates. Heterogeneity between studies was quantified using the *I* ^2^ statistic, with values of 25%–50% interpreted as low, 50%–75% as moderate, and greater than 75% as high heterogeneity [23]. For outcomes demonstrating high heterogeneity, a leave‐one‐out sensitivity analysis was conducted, in which each study was removed in turn to evaluate its impact on the overall effect estimate and identify any individual study exerting undue influence. Statistical significance was defined as a *p*‐value less than 0.05.

## 3 Results

### 3.1 Study Selection

The database search yielded 1721 records. After removing 751 duplicates, 970 unique articles remained for title and abstract screening. Of these, 886 were excluded for not meeting the eligibility criteria, and 84 full‐text articles were retrieved for further assessment. Following full‐text screening, 6 studies met the inclusion criteria and were incorporated into the final analysis [14, 15, 17, 18, 24, 25]. One crossover trial was excluded at this stage due to differences in study design, as it did not meet the eligibility criteria for parallel‐group RCTs [26]. The selection process is illustrated in the PRISMA flow diagram (Figure S1).

### 3.2 Study and Patient Characteristics

All included studies were RCTs published between 2023 and 2025. Each trial compared once‐weekly efsitora alfa with standard once‐daily insulin. Degludec was used as the comparator in four studies, while three trials employed glargine. Only one trial followed a crossover design. The mean duration of follow‐up across the included studies was 44.3 weeks. Continuous glucose monitoring (CGM) was performed using the Dexcom G6 device in most studies, with the exception of one trial that used Libre Pro and another that did not incorporate CGM. In total, 3967 participants were included in the pooled analysis, of whom 2144 received efsitora alfa. Overall, 2136 (53.8%) of the participants were male and 1831 (46.32%) were female. The mean age of the study population was 58.8 ± 10.5 years. Baseline characteristics of the included trials are summarised in Table 1.

**TABLE 1:** Study details and patient baseline characteristics of included RCTs.

Study	Year	Study duration (wks)	Continuous glucose‐monitoring device used	Study arms		Sample size		Age (Mean ± SD)		Males, *n* (%)		BMI (kg/m^2^)		Weight (kg)		Diabetes duration (years)		HbA1c (%)		Fasting plasma glucose (mg/dL)	
				Intervention	Control	Intervention	Control	Intervention	Control	Intervention	Control	Intervention	Control	Intervention	Control	Intervention	Control	Intervention	Control	Intervention	Control
Bue‐Valleskey et al.	2023	26	Libre Pro (blinded)	Efsitora	Degludec	129	135	57.4 ± 9.9	59.4 ± 9.1	71 (55)	76 (56.3)	32.2 ± 5.3	31.6 ± 5.5	91.3 ± 21.0	90.6 ± 19.6	10.6 ± 6.9	9.7 ± 6.0	8.1 ± 0.8	8.0 ± 0.8	169.7 ± 42.0	160.7 ± 36.7
Frias et al.	2023	32	Dexcom G6 (unblinded)	Efsitora	Degludec	132	132	59.6 ± 11.3	60.8 ± 10.0	62 (47)	67 (51)	32.4 ± 5.8	31.8 ± 5.7	88.1 ± 18.9	87.1 ± 20.7	14.1 ± 9.1	15.1 ± 8.0	8.0 ± 0.9	8.1 ± 0.9	141.7 ± 47.5	144.5 ± 51.0
Wysham et al.	2024	52	Dexcom G6 (unblinded)	Efsitora	Degludec	466	462	57.6 ± 10.6	57.3 ± 11.0	281 (60.3)	265 (57.4)	30.44 ± 5.85	30.72 ± 5.90	86.83 ± 20.53	86.12 ± 18.93	11.78 ± 7.54	11.42 ± 6.97	8.21 ± 0.96	8.23 ± 0.96	162.32 ± 45.79	165.13 ± 48.78
Philis‐Tsimikas et al.	2025	78	Dexcom G6 (unblinded)	Efsitora	Degludec	655	331	62.0 ± 10.4	61.0 ± 9.68	376 (57)	179 (54)	29.9 ± 5.72	30.33 ± 5.81	83.83 ± 18.65	84.73 ± 18.84	15.00 ± 8.54	14.70 ± 7.07	7.73 ± 0.97	7.70 ± 0.89	131.40 ± 41.23	131.67 ± 38.72
Blevins et al.	2025	26	Dexcom G6 (unblinded)	Efsitora	Glargine	365	365	58.3 ± 10.5	59.4 ± 10.5	172 (47)	189 (52)	31.85 ± 5.48	31.84 ± 5.48	87.8 ± 19.9	88.4 ± 19.7	16.6 ± 8.8	16.9 ± 9.0	8.36 ± 0.78	8.36 ± 0.80	148.3 ± 49.4	152.9 ± 54.7
Rosenstock et al.	2025	52	Not performed	Efsitora	Glargine	397	398	56.4 ± 10.0	56.2 ± 9.7	203 (51.1)	195 (49.0)	32.5 ± 5.8	31.3 ± 6.1	89.3 ± 19.2	85.5 ± 19.7	9.2 ± 6.6	9.6 ± 6.9	8.20 ± 0.91	8.27 ± 1.07	161 ± 48	161 ± 52

### 3.3 Results of Quality Assessment

Risk of bias assessment using the RoB 2.0 tool revealed that all domains were judged to be at low risk of bias across most domains. However, all studies were rated as having “some concerns” in domain 4 (measurement of the outcome), primarily due to limitations related to the blinding of outcome assessment and the reliance on objective measures that may still be subject to detection bias. No study was judged to be at high risk of bias in any domain. Detailed results of the bias assessment are presented in Figures S2 and S3.

### 3.4 Efficacy Outcomes

#### 3.4.1 Change in HbA1c (%)

**FIGURE 1:**
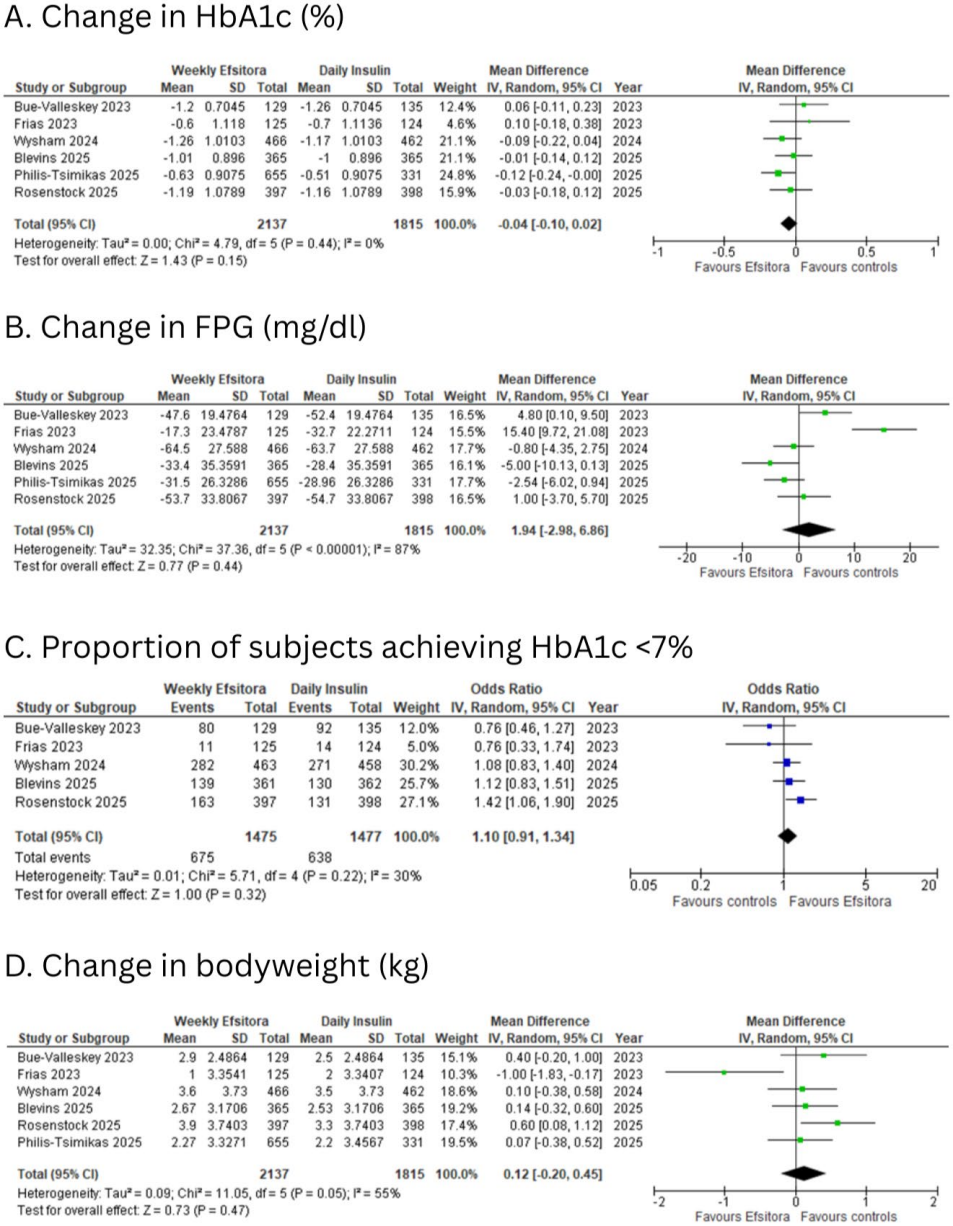
Forest plots for (A) change in HbA1c (%); (B) change in FPG (mg/dL); (C) proportion of subjects achieving HbA1c < 7%; (D) change in bodyweight (kg).

All included studies reported change in HbA1c from baseline. The reported glycated haemoglobin (HbA1c) change from baseline showed no statistically significant difference between the two treatment arms (MD: −0.04; 95% CI: −0.10 to 0.02; *I* ^2^: 0%; *p* = 0.15; Figure 1A).

The subgroup analysis based on comparator type showed no significant difference between groups; studies comparing efsitora with degludec (MD: −0.05; 95% CI: −0.14 to 0.05; *I* ^2^ = 31%; *p* = 0.33) and studies comparing efsitora with glargine (MD: −0.02; 95% CI: −0.12 to 0.08; *I* ^2^ = 0%; *p* = 0.71). The test for subgroup differences was not statistically significant (*p* = 0.68; Figure S7).

The subgroup analysis based on CGM use showed no significant difference between groups; studies in which CGM was used (MD: −0.04; 95% CI: −0.11 to 0.03; *I* ^2^ = 16%; *p* = 0.25) and the study without CGM use (MD: −0.03; 95% CI: −0.18 to 0.12; *I* ^2^ = not applicable; *p* = 0.70). The test for subgroup differences was not statistically significant (*p* = 0.88; Figure S11).

#### 3.4.2 Change in Fasting Plasma Glucose, FPG (mg/dL)

All included studies reported a change in FPG. There was no significant difference in the change in FPG between the efsitora and the control group (MD: 1.94 mg/dL; 95% CI: −2.98 to 6.86; *I* ^2^: 87%; *p* = 0.44; Figure 1B).

The subgroup analysis based on comparator type showed no significant difference between groups; studies comparing efsitora with degludec (MD: 3.92; 95% CI: −2.92 to 10.76; *I* ^2^ = 91%; *p* = 0.26) and studies comparing efsitora with glargine (MD: −1.91; 95% CI: −7.79 to 3.97; *I* ^2^ = 65%; *p* = 0.52). The test for subgroup differences was not statistically significant (*p* = 0.21; Figure S8).

The subgroup analysis based on CGM use showed no significant difference between groups; studies in which CGM was used (MD: 2.17; 95% CI: −3.79 to 8.13; *I* ^2^ = 89%; *p* = 0.47) and the study without CGM use (MD: 1.00; 95% CI: −3.70 to 5.70; *I* ^2^ = not applicable; *p* = 0.68). The test for subgroup differences was not statistically significant (*p* = 0.76) (Figure S12).

#### 3.4.3 Proportion of Subjects Achieving HbA1c < 7%

Five of the six included studies reported the proportion of patients achieving the glycemic target of HbA1c < 7%. The odds of participants achieving the glycemic target of HbA1c < 7% were similar for both treatments (OR: 1.10; 95% CI: 0.91 to 1.34; *I* ^2^: 30%; *p* = 0.32; Figure 1C).

#### 3.4.4 Change in Body Weight (kg)

All included studies reported a change in body weight. Treatment with weekly efsitora had a similar effect on body weight as daily insulin (MD: 0.12 kg; 95% CI: −0.20 to 0.45; *I* ^2^: 55%; *p* = 0.47; Figure 1D).

#### 3.4.5 Time in Range, TIR (% of Time Spent With Glucose 70–180 mg/dL)

**FIGURE 2:**
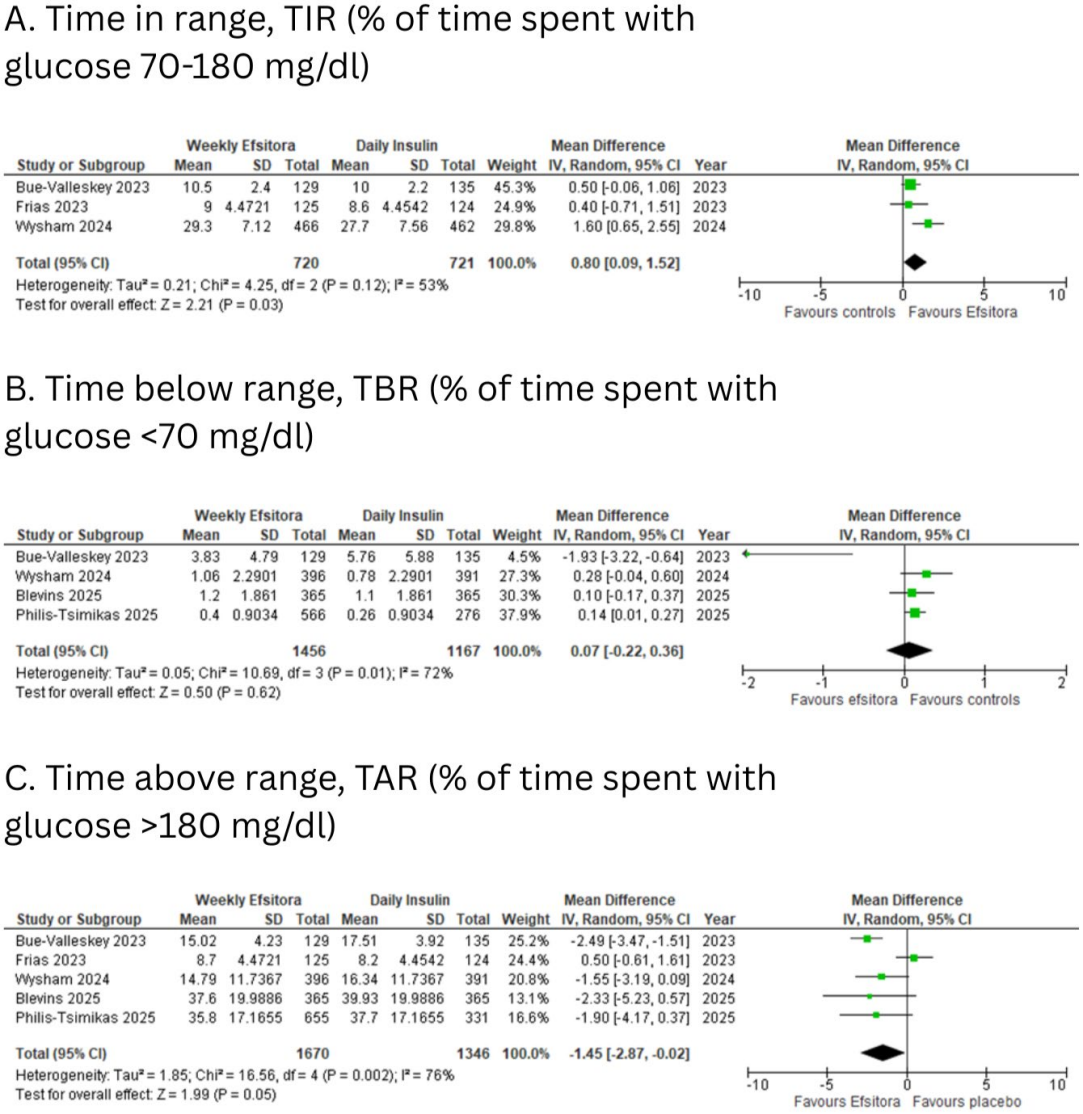
Forest plots for (A) time in range (% of time spent with glucose 70–180 mg/dL); (B) time below range (% of time spent with glucose < 70 mg/dL); (C) time above range (% of time spent with glucose > 180 mg/dL).

Three of the six included studies reported time in range metrics. Weekly efsitora was associated with a statistically significant increase in the percentage of time spent in the target glucose range (70–180 mg/dL) compared to daily insulin (MD: 0.80 percentage points; 95% CI: 0.09 to 1.52; *I* ^2^: 53%; *p* = 0.03; Figure 2A).

#### 3.4.6 Time Above Range, TAR (% of Time Spent With Glucose > 180 mg/dL)

Five of the six included studies reported time above range metrics. A statistically significant reduction in the percentage of time spent in time above range was observed with efsitora (MD: −1.45 percentage points; 95% CI: −2.87 to −0.02; *I* ^2^: 76%; *p* = 0.05; Figure 2B).

#### 3.4.7 Time Below Range (% of Time Spent With Glucose < 70 mg/dL)

Four of the six included studies reported time below range metrics. There was no significant difference in the percentage of time spent in hypoglycemia (MD: 0.07 percentage points; 95% CI: −0.22 to 0.36; *I* ^2^: 72%; *p* = 0.62; Figure 2C) between the two groups.

### 3.5 Safety Outcomes

#### 3.5.1 Treatment Emergent Adverse Events

**FIGURE 3:**
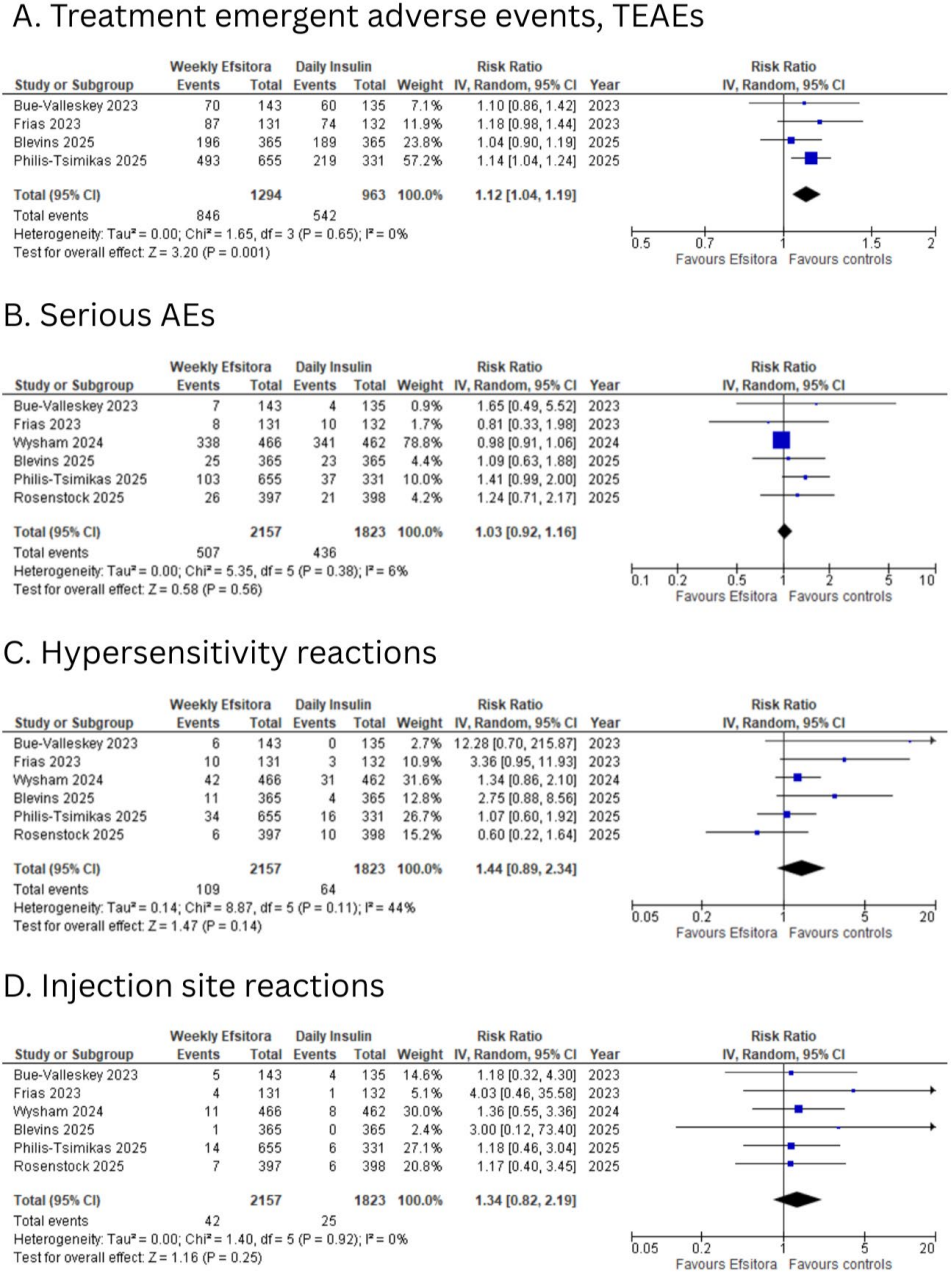
Forest plots for (A) treatment‐emergent adverse events (TEAEs); (B) serious adverse events; (C) hypersensitivity reactions; (D) injection site reactions.

Four of the six included studies reported treatment‐emergent adverse events. Pooled analysis revealed that participants in the weekly efsitora group had a higher risk compared to the daily insulin group (RR: 1.12; 95% CI: 1.04 to 1.19; *I* ^2^: 0%; *p* = 0.001; Figure 3A).

The subgroup analysis based on comparator type showed no significant difference between groups; studies comparing efsitora with degludec (RR: 1.14; 95% CI: 1.06 to 1.23; *I* ^2^ = 0%; *p* = 0.0007) and studies comparing efsitora with glargine (RR: 1.04; 95% CI: 0.90 to 1.19; *I* ^2^ = not applicable; *p* = 0.60). The test for subgroup differences was not statistically significant (*p* = 0.23) (Figure S9).

#### 3.5.2 Serious Adverse Events

All included studies reported total serious adverse events. Pooled analysis revealed no significant difference in the risk of serious adverse events (RR: 1.03; 95% CI: 0.92 to 1.16; *I* ^2^: 6%; *p* = 0.56; Figure 3B) between the two groups.

The subgroup analysis based on comparator type showed no significant difference between groups; studies comparing efsitora with degludec (RR: 1.09; 95% CI: 0.86 to 1.39; *I* ^2^ = 36%; *p* = 0.47) and studies comparing efsitora with glargine (RR: 1.16; 95% CI: 0.78 to 1.71; *I* ^2^ = 0%; *p* = 0.46). The test for subgroup differences was not statistically significant (*p* = 0.80) (Figure S10).

The subgroup analysis based on CGM use showed no significant difference between groups; studies in which CGM was used (MD: 1.06; 95% CI: 0.90 to 1.24; *I* ^2^ = 16%; *p* = 0.51) and the study without CGM use (MD: 1.24; 95% CI: 0.71 to 2.17; *I* ^2^ = not applicable; *p* = 0.45). The test for subgroup differences was not statistically significant (*p* = 0.58) (Figure S13).

#### 3.5.3 Hypersensitivity Reactions

All of the included studies reported data regarding hypersensitivity reactions. No statistically significant difference was observed in the risk of hypersensitivity reactions (RR: 1.44; 95% CI: 0.89 to 2.34; *I* ^2^: 44%; *p* = 0.14; Figure 3C) between the two groups.

#### 3.5.4 Injection Site Reactions

All included studies reported data regarding injection site reactions. No statistically significant difference was observed in injection site reactions (RR: 1.34; 95% CI: 0.82 to 2.19; *I* ^2^: 0%; *p* = 0.25; Figure 3D) between the two groups.

#### 3.5.5 Hypoglycemia Alerts

**FIGURE 4:**
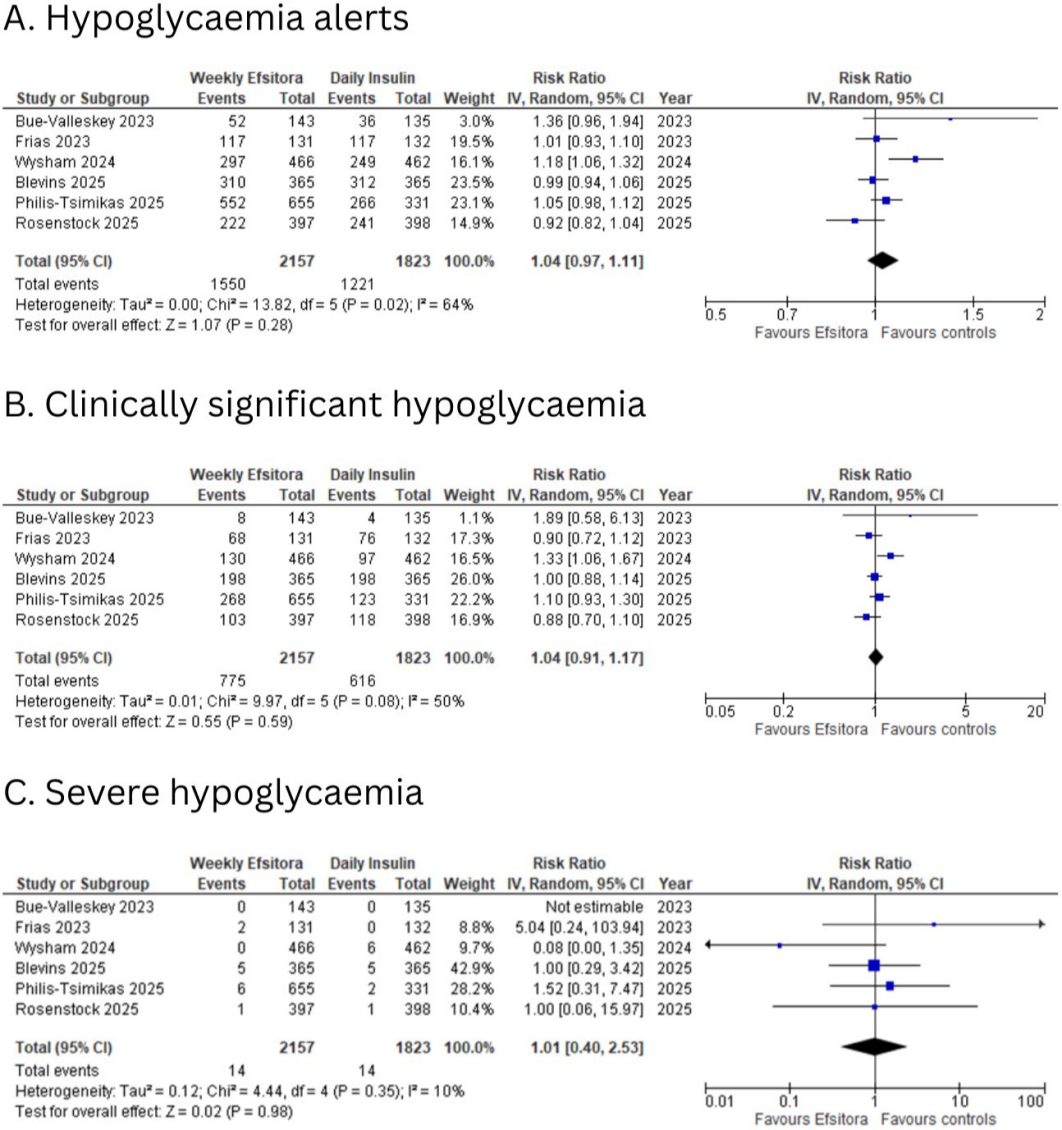
Forest plots for (A) hypoglycemia alerts; (B) clinically significant hypoglycemia; (C) severe hypoglycemia.

All included studies reported data regarding participants experiencing at least one hypoglycemic alert value. Pooled analysis showed no statistically significant difference (RR: 1.04; 95% CI, 0.97 to 1.11; *I* ^2^: 64%; *p* = 0.28; Figure 4A) between the two groups.

#### 3.5.6 Clinically Significant Hypoglycemia

All included studies reported data regarding participants experiencing at least one clinically significant hypoglycemic event. Pooled analysis showed no statistically significant difference (RR: 1.04; 95% CI, 0.91 to 1.17; *I* ^2^: 50%; *p* = 0.59; Figure 4B) between the two groups.

#### 3.5.7 Severe Hypoglycemia

All included studies reported data regarding participants experiencing at least one severe hypoglycemic event. Pooled analysis showed no statistically significant difference (RR: 1.01; 95% CI, 0.40 to 2.53; *I* ^2^: 10%; *p* = 0.98; Figure 4C) between the two groups.

### 3.6 Leave‐One‐Out Sensitivity Analysis

A leave‐one‐out sensitivity analysis was performed for outcomes demonstrating high heterogeneity. For the outcome of change in FPG, excluding Frias [24] reduced the heterogeneity from 87% to 58%. In both cases, the pooled estimate remained statistically non‐significant.

For the outcome of time above range, excluding Frias [24] reduced the heterogeneity from 76% to 0%, and the pooled effect remained statistically significant. For the same outcome, excluding either Bue‐Valleskey [14] reduced the heterogeneity to 61%. The pooled effect became non‐significant.

Plots for leave‐one‐out sensitivity analysis are provided in Figures S4–S6.

## 4 Discussion

In this systematic review and meta‐analysis of six RCTs including 3967 adults with type 2 diabetes, we compared once‐weekly efsitora alfa with standard once‐daily insulin. Overall, weekly efsitora provided similar reductions in HbA1c, fasting plasma glucose, and body weight, with comparable rates of patients achieving HbA1c targets. It also showed a favourable glycaemic profile improvement, reflected by increased time‐in‐range and reduced time‐above‐range, without increasing the time spent in hypoglycaemia. While serious adverse events and hypoglycaemic outcomes were comparable between the two groups, efsitora was associated with a higher incidence of treatment‐emergent adverse events.

Insulin efsitora alfa (also known as basal insulin Fc, BIF, or LY320950) is a novel fusion protein consisting of a single‐chain insulin analogue linked to the Fc domain of human IgG2 [13]. Incorporation of the Fc domain enables interaction with the neonatal Fc receptor (FcRn), facilitating recycling and thereby reducing degradation [12]. This design extends the circulating half‐life to approximately 15–16 days in humans, allowing once‐weekly administration while maintaining stable insulin exposure [27]. Although efsitora alfa exhibits around 100‐fold lower affinity for the insulin receptor compared with native insulin, it functions as a full agonist and effectively activates downstream signalling pathways responsible for glucose uptake and metabolism [12, 13, 28]. The reduced binding affinity and slower engagement are intentional, allowing for gradual and a more sustained insulin effect, which in turn minimizes peak‐to‐trough variability. Collectively, these pharmacokinetic and pharmacodynamics properties produce a nearly flat insulin activity profile that closely approximates physiological basal insulin secretion over the course of a week.

By reducing injection frequency, a weekly insulin has the potential to improve adherence and patient satisfaction [10, 29]. Supporting this, a survey‐based study in the US found that over 90% of patients with type 2 diabetes and their physicians preferred a once‐weekly insulin regimen compared with daily injections [30]. Multiple RCTs have evaluated once‐weekly efsitora against once‐daily insulin glargine or degludec, consistently demonstrating non‐inferior glycemic control in both type 1 and type 2 diabetes [15, 31]. The transition to a weekly insulin regimen may reflect outcomes already observed with once‐weekly GLP‐1 receptor agonists, which have demonstrated improved adherence and greater treatment satisfaction in both treatment‐naïve patients and those switching from daily therapy [32, 33, 34, 35, 36].

In the present meta‐analysis, once‐weekly efsitora achieved glycemic outcomes that were essentially equivalent to those observed with daily insulin. Both regimens produced comparable reductions in mean HbA1c, and the likelihood of attaining the recommended glycemic target (HbA1c < 7.0%) did not differ significantly. These findings are in line with prior evidence supporting the clinical equivalence of weekly and daily basal insulins. For example, Karakasis et al. reported no meaningful difference in HbA1c reduction between weekly and daily formulations, and Wang et al. found the estimated mean difference in HbA1c reduction to be negligible [37, 38]. An updated synthesis by Xue et al. similarly noted only a modest advantage for weekly analogues with a slightly higher odds of achieving HbA1c < 7% in patients treated with once‐weekly efsitora or icodec [39]. Additional meta‐analyses focusing specifically on efsitora, including those by Raja et al. and Dutta et al., have also demonstrated non‐inferiority of weekly formulations compared to daily insulin in terms of HbA1c reduction and target attainment [16, 40]. These consistent findings across multiple independent syntheses reinforce the conclusion that the less frequent dosing schedule of efsitora does not compromise glycemic efficacy and may therefore represent a viable alternative to conventional daily insulin.

Similar conclusions were reached in broader reviews examining once‐weekly basal insulins in both type 1 and type 2 diabetes. Altobaishat et al. reported that while once‐weekly insulins were associated with higher rates of injection‐site reactions and treatment‐emergent adverse events, they achieved HbA1c and fasting glucose control comparable to daily regimens [41]. Abuelazm et al. focused specifically on insulin icodec and found equivalent HbA1c and fasting plasma glucose reductions compared to daily basal insulins, while Saleem et al. additionally reported a modest improvement in time‐in‐range with once‐weekly icodec compared with daily insulin analogues [42, 43]. While icodec has been extensively studied in prior reviews, an efsitora‐specific synthesis was needed to clarify whether these observations are consistent across different once‐weekly insulin analogues. The present analysis therefore extends existing literature by incorporating the most recent efsitora trials and CGM data, allowing a more granular evaluation of day‐to‐day glycaemic stability.

We observed no significant difference in fasting plasma glucose (FPG) reductions between once‐weekly efsitora and daily insulin, a result consistent with previous meta‐analyses [16, 40]. Likewise, Soetedjo et al. found that weekly insulin icodec achieved comparable FPG control relative to daily insulins [44]. Interestingly, their analysis also demonstrated a greater reduction in HbA1c with icodec, suggesting that certain structural modifications may translate into incremental improvements in long‐term glycemic control. From a pharmacokinetic standpoint, the two analogues achieve extended duration through distinct mechanisms: efsitora is an Fc‐domain fusion protein with a molecular weight of 64.1 kDa, limiting renal clearance and producing a flat, sustained exposure profile, whereas icodec employs a C20 fatty diacid side chain and amino acid substitutions to enhance albumin binding, reduce receptor affinity, and prolong its half‐life beyond 8 days [45]. Collectively, these findings highlight that both efsitora and icodec provide effective once‐weekly insulin coverage without compromising fasting glucose regulation, while icodec may offer modest additional benefit in HbA1c lowering.

Our analysis demonstrated comparable body weight changes between once‐weekly efsitora and daily insulin. Although insulin therapy is well known to promote weight gain, primarily through its anabolic effects and compensatory eating following hypoglycemia, such increases were not observed with the weekly regimen [46]. This finding is consistent with recent meta‐analyses, which likewise reported no excess weight gain with efsitora compared to daily insulins [16]. In their subgroup analysis, Raja et al. further demonstrated that once‐weekly insulin icodec was associated with significant weight gain, whereas efsitora showed no significant difference compared with daily regimens [16]. The absence of additional weight burden is clinically meaningful, as weight gain remains a major barrier to insulin initiation and long‐term adherence [46, 47, 48]. A plausible explanation lies in its flat and sustained exposure profile, which may reduce glycemic variability, hypoglycemia, and the subsequent defensive caloric intake that often drives weight gain with conventional insulin regimens [13]. These observations suggest that once‐weekly efsitora maintains glycemic efficacy without aggravating the well‐recognised problem of insulin‐associated weight gain, thereby supporting its potential role as a more acceptable alternative for patients concerned about treatment‐related weight effects.

With respect to CGM parameters, we found that efsitora was associated with a significant increase in time‐in‐range (TIR) and a reduction in time‐above‐range (TAR) compared with daily insulin, without prolonging time‐below‐range (TBR). Since TIR (% of time spent with glucose 70–180 mg/dL) is increasingly recognized as a robust indicator of glycemic control, closely linked to microvascular complications and complementary to HbA1c, these findings suggest that weekly insulin may offer superior day‐to‐day stability without additional hypoglycemia risk [49, 50]. The International Consensus further recommends maintaining TIR above 70% for most patients with diabetes, underscoring the clinical relevance of this endpoint [50, 51]. However, the absolute difference we observed of approximately 0.8 percentage points is below the 5% increase generally regarded as clinically meaningful, as highlighted by the International Consensus, which noted that each incremental 5% rise in TIR is associated with significant clinical benefit [52]. In contrast, Dutta et al. reported no significant differences in these measures between weekly and daily regimens, which may be attributable to the exclusion of more recent trials in their review [40]. The QWINT‐3 trial, for example, demonstrated a modest but statistically significant improvement in TIR with efsitora (62.8%) compared with degludec (61.3%) [18]. Similarly, Xue et al., when analyzing both type 1 and type 2 diabetes, found no overall TIR difference; yet subgroup analyses revealed significantly higher TIR in insulin‐naïve type 2 patients, suggesting that weekly dosing may confer selective benefits depending on population characteristics [39]. Time‐above‐range also tended to be lower with weekly insulin; Abunada et al. reported no excess time spent above 250 mg/dL with efsitora, emphasizing its capacity to reduce hyperglycemic excursions [53]. Importantly, while our findings did not show any increase in TBR, Raja et al. reported a very small but statistically significant increase in time spent below range, though the clinical impact of this marginal difference remains uncertain [16].

When evaluating safety outcomes, particularly hypoglycemia, our meta‐analysis found that hypoglycemia rates did not differ significantly between efsitora and daily insulin. We observed comparable proportions of patients experiencing level 1 (alert), level 2 (clinically significant), and level 3 (severe) hypoglycemia in both groups. This finding is generally consistent with existing data. For example, Dutta et al. reported no difference in overall or severe hypoglycemia incidence in T2D patients on efsitora versus degludec, with even a lower risk of nocturnal hypoglycemia in the efsitora arm [40]. Likewise, Wang et al. found weekly analogues did not increase hypoglycemia risk [38]. Some analyses have noted a slight rise in mild hypoglycemia (level 1) with weekly insulin: Xue et al. found a higher odds of level 1 events (OR 1.42) with once‐weekly analogues, though level 2/3 events were similar [39]. Overall, the consensus is that hypoglycemia, especially severe events, is not substantially increased with weekly regimens, and efsitora's risk of hypoglycemia appears comparable to daily insulin.

Safety profiles were generally comparable between efsitora and daily insulin. In our analysis, SAEs and injection‐site or hypersensitivity reactions occurred at similar rates across groups, although overall TEAEs were modestly higher with efsitora, a finding consistent with the results of Raja et al. [16]. Dutta et al. [40] reported equivalent rates of SAEs, injection‐site reactions, and hypersensitivity in patients with type 2 diabetes, whereas in type 1 diabetes, the incidence of TEAEs, SAEs, and injection‐site events was slightly higher with efsitora. Importantly, the excess TEAEs observed with efsitora were largely mild or moderate (e.g., nasopharyngitis, injection‐site discomfort) and did not translate into excess treatment discontinuations or clinically significant safety concerns. Furthermore, injection‐site and hypersensitivity reactions have not emerged as a prominent issue in trials, with randomised studies and pooled analyses consistently showing low rates, comparable to those seen with daily insulin. Taken together, the evidence supports that once‐weekly efsitora is as safe as daily insulin with respect to major safety outcomes, with slightly higher adverse events limited to expected and generally tolerable effects.

The high heterogeneity observed in fasting plasma glucose (FPG) outcomes was substantially reduced in leave‐one‐out sensitivity analyses when the Frias et al. [24] trial was excluded. This reduction is likely explained by the trial's unequal fasting glucose targets, where insulin degludec was titrated to a stricter goal (≤ 100 mg/dL) compared to efsitora (≤ 120 mg/dL), resulting in comparatively larger FPG reductions in the degludec arm. Likewise, heterogeneity in time‐above‐range (TAR) outcomes decreased markedly on exclusion of either the Frias et al. or Bue‐Valleskey et al. trials, reaching zero in the former case [14, 24]. Such findings underscore the role of trial design and population characteristics in driving variability. In particular, the inclusion of insulin‐naïve participants in Bue‐Valleskey et al. may have contributed, as these individuals often display different glycemic responses than the insulin‐experienced population in Frias et al. Other contributing factors may include differences in titration algorithms, baseline glycemic control, and fasting glucose targets across trials, each of which can influence glycemic variability and time spent above range.

## 5 Limitations

This analysis has several limitations. The included trials were of relatively limited duration, which constrains evaluation of very long‐term outcomes. Most were open‐label, introducing potential performance and ascertainment bias. High heterogeneity was observed in certain outcomes, likely stemming from differences in fasting glucose targets, titration protocols, baseline regimens, and study methodologies; although random‐effects models were applied, some residual variability may persist. Furthermore, most evidence derives from manufacturer‐sponsored RCTs conducted under controlled conditions, which may not fully reflect real‐world clinical practice. Finally, data on long‐term safety, adherence, cost‐effectiveness, and diabetes‐related complications remain sparse.

## 6 Conclusion

Our meta‐analysis of six RCTs involving almost 4000 adults with type 2 diabetes demonstrates that once‐weekly insulin efsitora alfa provides glycemic efficacy comparable to once‐daily insulin, with similar reductions in HbA1c, fasting plasma glucose, body weight, and achievement of HbA1c targets. Weekly efsitora was associated with favourable continuous glucose monitoring outcomes, including increased time‐in‐range and reduced time‐above‐range, without increasing time spent in hypoglycemia. While serious adverse events, hypersensitivity, injection site reactions, and hypoglycemic episodes of all severities occurred at rates similar to daily insulin, efsitora was associated with a higher incidence of treatment‐emergent adverse events. Future long‐term studies are needed to establish its durability, safety profile, and impact on diabetes‐related complications in real‐world populations.

## Author Contributions

Conceptualization, data curation, and project administration were carried out by M.A. (Mushood Ahmed) and E.Z. Supervision was carried out by E.Z., M.A. (Mushood Ahmed), R.A., and A.N. Formal analysis of data was carried out by H.M., M.A. (Moaz Alowami), and E.Z. Formal analysis, methodology, and software were carried out by H.M., M.A. (Moaz Alowami), T.S.H., K.N.Z., and E.Z. Writing the original draft was carried out by H.M., M.A. (Moaz Alowami), T.S.H., K.N.Z., Y.U., A.M.A., A.A., O.I., and S.F.A. Writing, reviewing, and editing were carried out by E.Z., M.A. (Mushood Ahmed), M.F., R.A., and A.N. Visualisation and validation were carried out by M.A. (Mushood Ahmed) and E.Z.

## Ethics Statement

The authors have nothing to report.

## Consent

The authors have nothing to report.

## Conflicts of Interest

The authors declare no conflicts of interest.

## Supporting information


**Appendix S1:** edm270126‐sup‐0001‐AppendixS1.zip.

## Data Availability

All data generated or analyzed during this study are included in this article. Further inquiries can be directed to the corresponding author.
