# Supplementary material for: Once‐Weekly Insulin Efsitora Alfa Versus Once Daily Insulin in Patients With Type 2 Diabetes: A Systematic Review and Meta‐Analysis
Source: Endocrinol Diabetes Metab. 2025 Oct 28;8(6):e70126. doi: 10.1002/edm2.70126 (PMC12568379; doi:10.1002/edm2.70126)
Supplement: Supplementary file 1 — Appendix S1: edm270126‐sup‐0001‐AppendixS1.zip. [file EDM2-8-e70126-s001.zip › edm270126-sup-0001-TableS1.docx]

Table : Assessment of certainty of evidence

**Once-Weekly Insulin Efsitora Alfa Versus Once Daily Insulin in patients with Type 2 Diabetes**

| **Certainty assessment** | | | | | | |
| --- | --- | --- | --- | --- | --- | --- |
| **Participants (studies)** | **Risk of bias** | **Inconsistency** | **Indirectness** | **Imprecision** | **Publication bias** | **Overall certainty of evidence** |
| **Change in HbA1c** | | | | | | |
| 3952 (6 RCTs) | not serious | not serious | not serious | serious | undetected | ⨁⨁⨁◯^a^ Moderate |
| **Change in FPG** | | | | | | |
| 3952 (6 RCTs) | not serious | serious | not serious | serious | undetected | ⨁⨁◯◯^b^ Low |
| **Proportion of subjects achieving HbA1c <7%** | | | | | | |
| 2952 (5 RCTs) | not serious | not serious | not serious | serious | undetected | ⨁⨁⨁◯^c^ Moderate |
| **Change in bodyweight** | | | | | | |
| 3952 (6 RCTs) | not serious | not serious | not serious | serious | undetected | ⨁⨁⨁◯^d^ Moderate |
| **Time in range** | | | | | | |
| 1441 (3 RCTs) | not serious | not serious | not serious | not serious | undetected | ⨁⨁⨁⨁ High |
| **Time below range** | | | | | | |
| 2623 (4 RCTs) | not serious | not serious | not serious | serious | undetected | ⨁⨁⨁◯^e^ Moderate |
| **Time above range** | | | | | | |
| 3016 (5 RCTs) | not serious | serious | not serious | not serious | undetected | ⨁⨁⨁⨁ High |
| **TEAEs** | | | | | | |
| 2257 (4 RCTs) | not serious | not serious | not serious | not serious | undetected | ⨁⨁⨁⨁ High |
| **Serious AEs** | | | | | | |
| 3980 (6 RCTs) | not serious | not serious | not serious | serious | undetected | ⨁⨁⨁◯^f^ Moderate |
| **Hypersensitivity reactions** | | | | | | |
| 3980 (6 RCTs) | not serious | not serious | not serious | serious | undetected | ⨁⨁⨁◯^g^ Moderate |
| **Injection site reactions** | | | | | | |
| 3980 (6 RCTs) | not serious | not serious | not serious | serious | undetected | ⨁⨁⨁◯^h^ Moderate |
| **Hypoglycemia alerts** | | | | | | |
| 3980 (6 RCTs) | not serious | not serious | not serious | not serious | undetected | ⨁⨁⨁⨁ High |
| **Clinically significant hypoglycemia** | | | | | | |
| 3980 (6 RCTs) | not serious | not serious | not serious | not serious | undetected | ⨁⨁⨁⨁ High |
| **Severe hypoglycemia** | | | | | | |
| 3980 (6 RCTs) | not serious | not serious | not serious | not serious | undetected | ⨁⨁⨁⨁ High |

a Downgraded one level for imprecision due to wide confidence intervals crossing the line of no effect.

b Downgraded two levels for inconsistency and imprecision due to substantial heterogeneity (I² > 50%) and wide confidence intervals.

c Downgraded one level for imprecision due to wide confidence intervals and limited number of events.

d Downgraded one level for imprecision due to wide confidence intervals.

e Downgraded one level for imprecision due to wide confidence intervals.

f Downgraded one level for imprecision due to wide confidence intervals and small number of events.

g Downgraded one level for imprecision due to wide confidence intervals and low event rates.

h Downgraded one level for imprecision due to wide confidence intervals and small number of events.
