# Supplementary material for: Once‐Weekly Insulin Efsitora Alfa Versus Once Daily Insulin in Patients With Type 2 Diabetes: A Systematic Review and Meta‐Analysis
Source: Endocrinol Diabetes Metab. 2025 Oct 28;8(6):e70126. doi: 10.1002/edm2.70126 (PMC12568379; doi:10.1002/edm2.70126)

**Table S1: Search strategy**

| **Database** | **Search string** | **Records** |
| --- | --- | --- |
| PubMed/MEDLINE | ("Diabetes Mellitus, Type 2"[MeSH Terms] OR "type 2 diabetes"[Title/Abstract] OR "T2D"[Title/Abstract] OR "non-insulin dependent diabetes"[Title/Abstract] OR "non insulin dependent diabetes"[Title/Abstract]) AND ("Insulin, Long-Acting"[MeSH Terms] OR "long-acting insulin"[Title/Abstract] OR "long acting insulin"[Title/Abstract] OR "insulin efsitora alfa"[Title/Abstract] OR "LY3209590"[Title/Abstract] OR "insulin Fc"[Title/Abstract] OR "basal insulin Fc"[Title/Abstract] OR "insulin BIF"[Title/Abstract] OR "once-weekly insulin"[Title/Abstract] OR "once weekly insulin"[Title/Abstract] OR "once-weekly basal insulin"[Title/Abstract] OR "once weekly basal insulin"[Title/Abstract]) AND ("Randomized Controlled Trial"[Publication Type] OR "randomized trial"[Title/Abstract] OR "randomized controlled trial"[Title/Abstract] OR "randomised controlled trial"[Title/Abstract] OR "RCT"[Title/Abstract]) | 879 |
| Google Scholar | ("insulin efsitora" OR "LY3209590" OR "basal insulin Fc" OR "once-weekly basal insulin" OR "once-weekly insulin") AND ("type 2 diabetes" OR "type 2 diabetes mellitus) AND ("randomized controlled trial" OR "randomized trial" OR RCT) | 658 |
| Cochrane Library | ("type 2 diabetes" OR T2D OR "non-insulin dependent diabetes" OR "non insulin dependent diabetes")  AND ("long-acting insulin" OR "long acting insulin" OR "insulin efsitora alfa" OR LY3209590 OR "insulin Fc" OR "basal insulin Fc" OR "insulin BIF" OR "once-weekly insulin" OR "once weekly insulin" OR "once-weekly basal insulin" OR "once weekly basal insulin")  AND ("randomized controlled trial" OR "randomised controlled trial" OR "randomized trial" OR RCT) | 184 |

**Figure S1: PRISMA Flow Diagram**


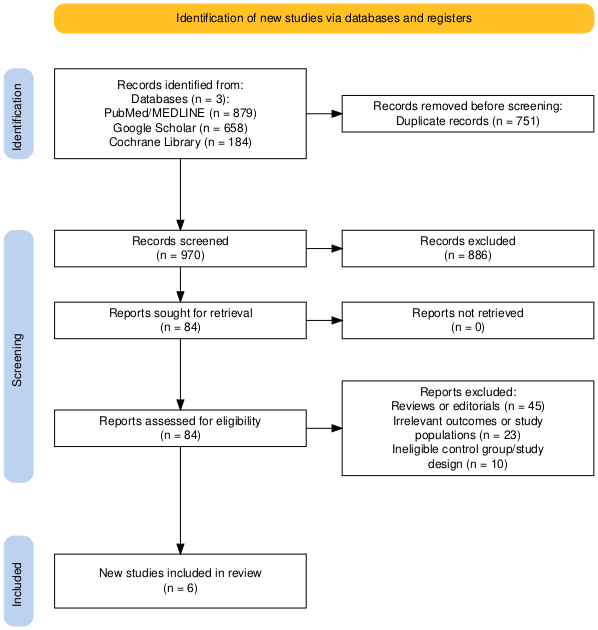


**Figure S2: Traffic light plot RoB 2 tool for risk of bias assessment**


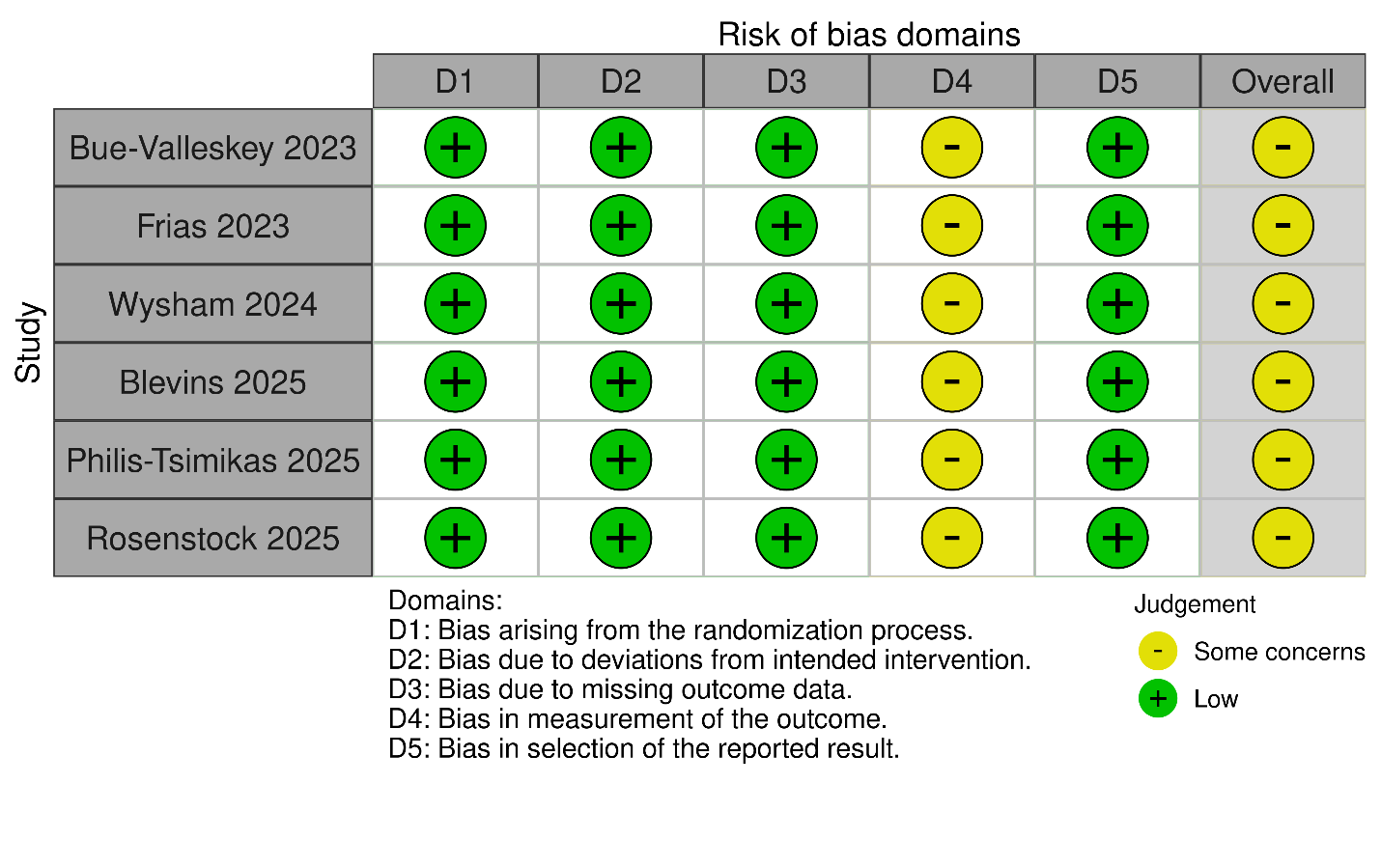


**Figure S3: Summary plot RoB 2 tool for risk of bias assessment**


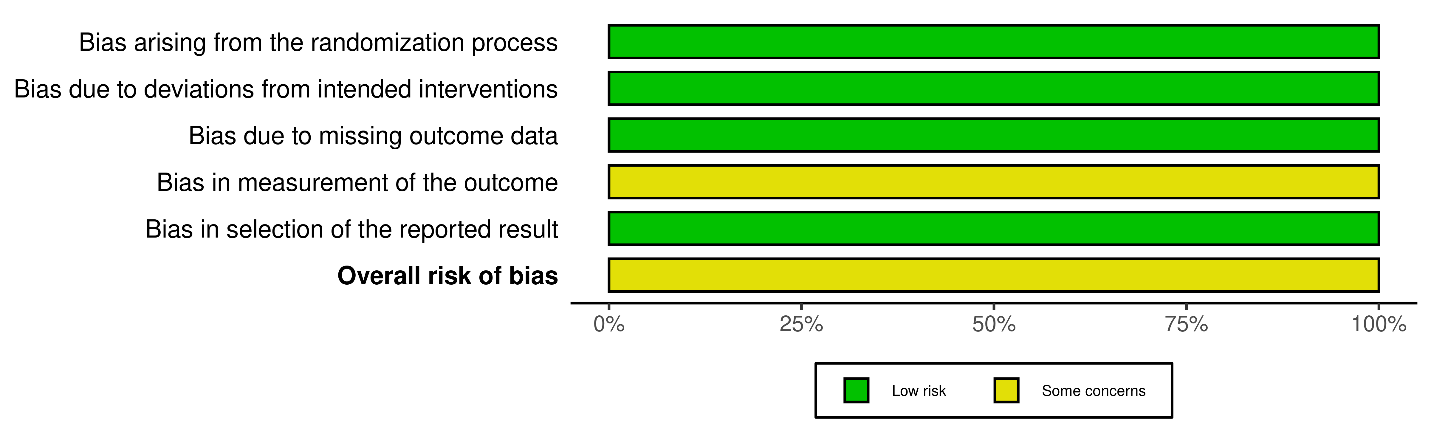


**Figure S4: Leave-one-out analysis for change in FPG, excluding Frias 2023**

**
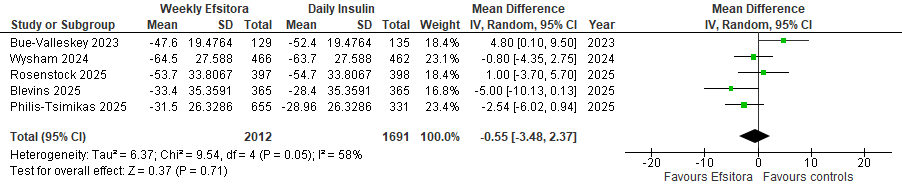
**

**Figure S5: Leave-one-out analysis for time above range, excluding Frias 2023**

**
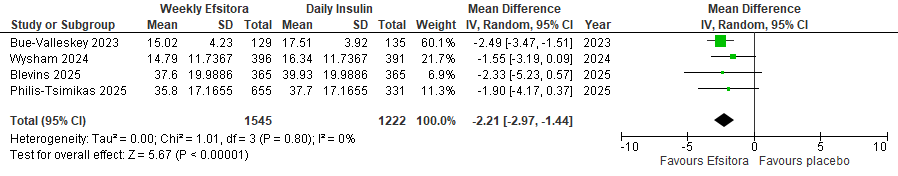
**

**Figure S6: Leave-one-out analysis for time above range, excluding Bue-Valleskey 2023**

**
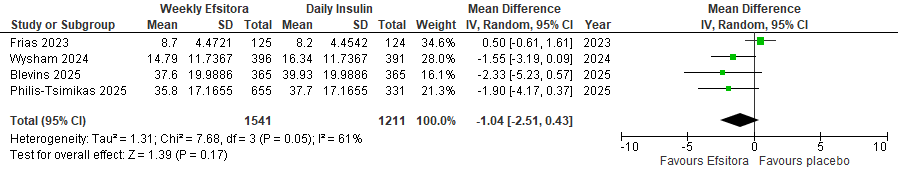
**

**Figure S7: Subgroup analysis based on the comparator type, for change in HbA1c**


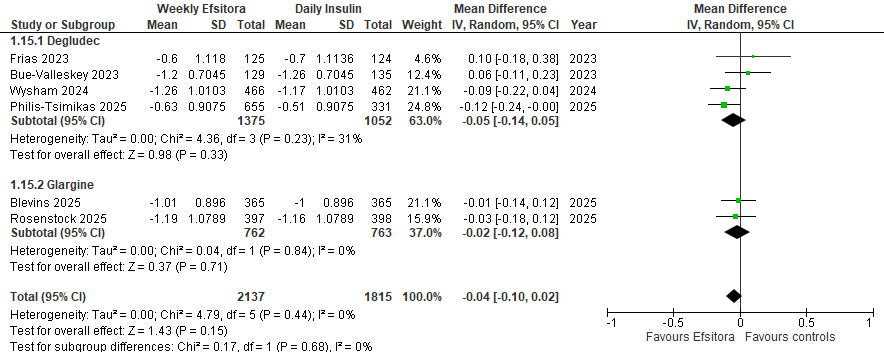


**Figure S8: Subgroup analysis based on the comparator type, for change in FPG**

**
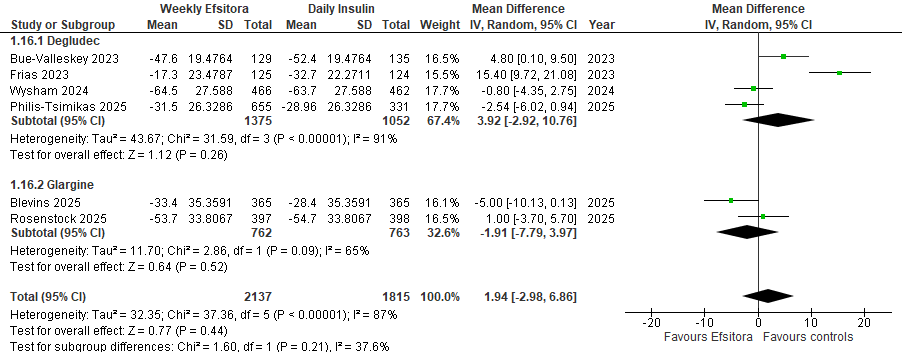
**

**Figure S9: Subgroup analysis based on the comparator type, for TEAEs**


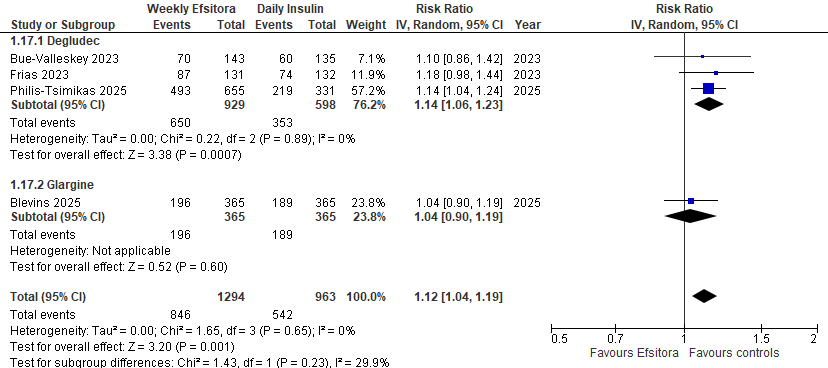


**Figure S10: Subgroup analysis based on the comparator type, for serious AEs**


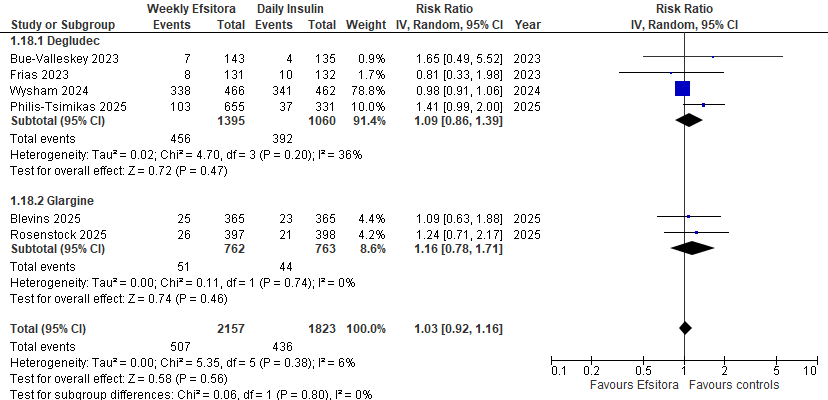


**Figure S11: Subgroup analysis based on the use of CGM, for change in HbA1c**


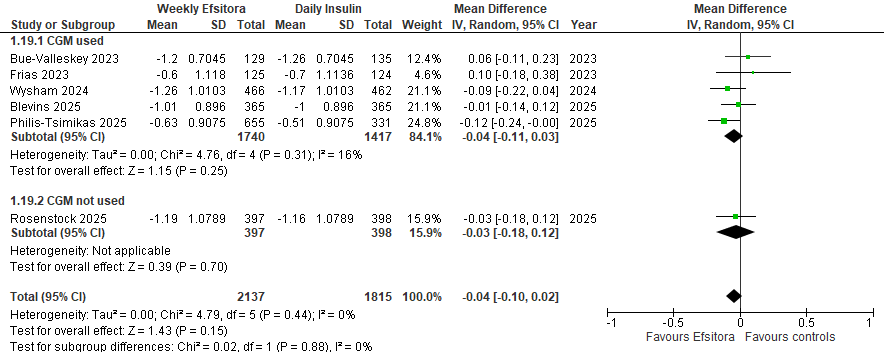


**Figure S12: Subgroup analysis based on the use of CGM, for change in FPG**

**
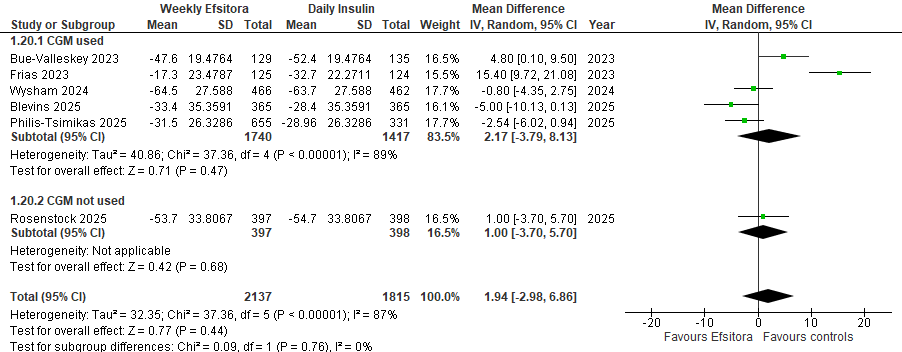
**

**Figure S13: Subgroup analysis based on the use of CGM, for serious AEs**


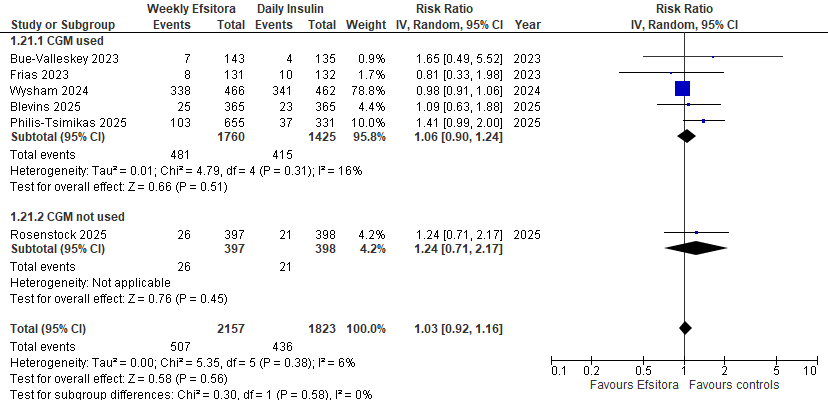

Supplement: Supplementary file 1 — Appendix S1: edm270126‐sup‐0001‐AppendixS1.zip. [file EDM2-8-e70126-s001.zip › edm270126-sup-0002-AppendixS1.docx]
